# *Pimpla* Fabricius, 1804 (Ichneumonidae, Pimplinae) from Uruguay: a replacement name, new records, and an identification key to the species

**DOI:** 10.3897/zookeys.1007.56328

**Published:** 2020-12-30

**Authors:** Diego G. Pádua, Daniell R. R. Fernandes, Ilari E. Sääksjärvi

**Affiliations:** 1 Programa de Pós-Graduação em Entomologia, Instituto Nacional de Pesquisas da Amazônia, Av. André Araújo, 2936, Petrópolis, 69067-375, Manaus, Amazonas, Brazil Programa de Pós-Graduação em Entomologia, Instituto Nacional de Pesquisas da Amazônia Manaus Brazil; 2 Biodiversity Unit, Zoological Museum, University of Turku, FIN-20014, Turku, Finland University of Turku Turku Finland

**Keywords:** Faunistics, homonymy, neotropics, parasitoids, parasitoid wasps, South America, taxonomy

## Abstract

We report new faunistic records of *Pimpla* Fabricius, 1804 from Uruguay. The following species are reported from the country for the first time: *P. albomarginata* Cameron, 1846, *P. caerulea* Brullé, 1846, *P. perssoni* Gauld, 1991, and *P. semirufa* Brullé, 1846. In addition, we propose a replacement name for *Pimpla rufipes* Brullé, 1846 and provide diagnosis, digital images, and an identification key for all the *Pimpla* species known to occur in Uruguay.

## Introduction

The parasitoid wasp family Ichneumonidae (Darwin wasps) is among the largest animal families anywhere on Earth (Klopfstein et al. 2019). It is taxonomically challenging, and many species are either rare or at least rarely collected. One exception is the genus *Pimpla* Fabricius, 1804 (Pimplinae, Pimplini). It is composed of moderately large (in tropical regions), often colorful species which are abundant in many entomological collections (Townes 1969; Porter 1970).

With over 200 valid species (Yu et al. 2016; Watanabe and Matsumoto 2019), this genus is among the largest genera within the subfamily Pimplinae. The species of *Pimpla* are known to be idiobiont endoparasitoids of prepupae and pupae of Lepidoptera (Gauld 1991). The genus is characterized by simple and large tarsal claws (females), the straight apex of the ovipositor, a weakly concave internal margin of the compound eye in front of the antennal insertion (Gauld et al. 1998), and mid tarsomere IV medioventrally with a longitudinal band of fine hair (an autapomorphy) (Gauld et al. 2002).

On account of the taxonomical works of Charles C. Porter in South America (Porter 1970) and Ian D. Gauld in Central America (Gauld 1991; Gauld et al. 1998), the genus is one of the best-known Darwin wasp genera in the Neotropical region. Porter (1970) reported 35 (21 of them new) species from South America and Gauld (1991) and Gauld et al. (1998) found 17 (six of them new) species in Costa Rica.

The aim of this paper is to provide new records of *Pimpla* from Uruguay. In addition, we provide diagnosis, high-quality layer-stacked photographs, and an identification key for the species currently known from the country. This work is part of a series of articles reporting new *Pimpla* records from South America. This work was started by Pádua et al. (2019).

## Material and methods

### Study area

The field sampling was conducted in four locations in the municipality of Castillos, Rocha Department, Uruguay, between December 2014 and December 2016 (see Fernandes et al. 2019).

### Specimens studied

*Pimpla* specimens were collected by Malaise trapping, and the specimens are deposited in the Invertebrate Collection of Instituto Nacional de Pesquisas da Amazônia (INPA; curator: Marcio L. Oliveira).

### Morphology and distribution

General morphological terminology follows that of Gauld (1991). New distributional records are indicated with an asterisk (*).

### Photographs

Digital images were taken using a Leica DMC4500 digital camera attached to a Leica M205A stereomicroscope and combined using the software Leica Application Suite V4.10.0. The final images were edited in Adobe Photoshop.

### Abbreviations

**BMNH**Natural History Museum, London, UK;

**IML**Institute Miguel Lillo, Tucumám, Argentina;

**MNCR**Museo Nacional de Costa Rica, San José, Costa Rica;

**MNHN**Muséum national d’Histoire naturelle, Paris, France.

### Distribution maps

The distribution maps were created using SimpleMappr online software (Shorthouse 2010).

### Key to the Uruguayan species of *Pimpla* Fabricius, 1804

**Table:** 

1	Female	**2**
–	Male (the male of *P. cyanipennis* Brullé, 1846 is unknown)	**9**
2	Mesosoma and metasoma metallic blue (Fig. 2A)	***P. caerulea* Brullé, 1846**
–	Mesosoma and metasoma black, brown, yellow, reddish, or a combination of these colours (Figs 1A, 3A, 5A, 6A, 8A, 9A, 10A)	**3**
3	Fore wing hyaline, with an apical darkened area (Fig. 8A); malar space 0.3–0.4 times as long as basal width of mandibles	***P. perssoni* Gauld, 1991**
–	Fore wing without an apical darkened area (Figs 1A, 3A, 5A, 6A, 9A, 10A); malar space > 0.6 times as long as basal width of mandibles	**4**
4	Laterotergite V < 1.7 times as long as wide (Figs 5E, 6E, 9E)	**5**
–	Laterotergite V > 2.1 times as long as wide (Figs 1E, 8E, 10E)	**8**
5	Metasoma entirely reddish (Fig. 5A)	***P. golbachi* (Porter, 1970)**
–	Metasoma entirely black or reddish with tergites VI+ black (Figs 3A, 9A)	**6**
6	Ovipositor > 1.7 times as long as hind tibia; meso- and metacoxa black (Figs 3A, C, 4A, C)	***P. cyanipennis* Brullé, 1846**
–	Ovipositor < 1.6 times as long as hind tibia; meso- and metacoxa reddish brown (Figs 6A, 9A)	**7**
7	Metasoma black (Fig. 6A)	***P. patirrufa* nom. nov.**
–	Metasoma reddish with tergites V+ or VI+ black (Fig. 9A)	***P. semirufa* Brullé, 1846**
8	Dorsal valve of ovipositor apically with teeth (Fig. 10F)	***P. tomyris* Schrottky, 1902**
–	Dorsal valve of ovipositor apically without teeth (Fig. 1F)	***P. albomarginata* Cameron, 1886**
9	Mesosoma and metasoma with a metallic blue (Fig. 2B)	***P. caerulea* Brullé, 1846**
–	Mesosoma and metasoma black, brown, yellow, reddish, or a combination of these colours (Figs 1B, 5B, 6B, 8B, 9B, 10B)	**10**
10	Fore wing hyaline with an apical darkened area (Fig. 8B)	***P. perssoni* Gauld, 1991**
–	Fore wing without an apical dark area (Figs 1B, 5B, 6B, 9B, 10B)	**11**
11	Metasomal tergites with fine punctures (Figs 1D, 10D)	**12**
–	Metasomal tergites with strong punctures (Figs 5D, 6D, 9D)	**13**
12	Mesosoma reddish with profuse white marks (Fig. 1B); metasoma black and white banded (Fig. 1B)	***P. albomarginata* Cameron, 1886**
–	Mesosoma shining black with variable patterning of yellow markings on pronotum, tegula, scutellum, postscutellum and propodeum (a pair of elliptic blotches) (Fig. 10B); metasoma reddish brown, with a pair of large yellow blotches laterally on tergites I–IV (Fig. 10B)	***P. tomyris* Schrottky, 1902**
13	Mesosoma entirely shining black (Fig. 6B)	***P. patirrufa* nom. nov.**
–	Mesosoma black with hind corners of meso- and metapleuron brown and tegula white or shining black with lower hind corner of mesopleuron brown, and metapleuron red with a little black staining along front margin (Figs 5B, 9B)	**14**
14	Metasoma reddish with tergite VI+ black (Fig. 9B)	***P. semirufa* Brullé, 1846**
–	Metasoma entirely reddish (Fig. 5B)	***P. golbachi* (Porter, 1970)**

## Faunistics and taxonomy

### Pimpla

Taxon classificationAnimaliaHymenopteraIchneumonidae

Fabricius, 1804

8FDE33D9-5991-5D27-A966-01AF77F6BA37

Pimpla Fabricius, 1804: 112. Type species: Ichneumon instigator Fabricius (= Ichneumon hypochondriaca Retzius), by subsequent designation (Opinion 159, International Commission on Zoological Nomenclature 1945: 282).
Coccygomimus Saussure, 1892: pl. 14, fig. 12. Type species: Coccygomimus madecassus Saussure, by monotypy.
Habropimpla Cameron, 1900: 96. Type species: Habropimpla bilineata Cameron, by monotypy.
Lissotheronia Cameron, 1905: 139. Type species: Lissotheronia flavipes Cameron, by monotypy.
Phytodiaetoides Morley, 1913: 221. Type species: Phytodiaetoides megaera Morley = Pimpla flavipalpis, by original designation.
Pimplidea Viereck, 1914: 117. Type species: Pimpla pedalis Cresson, by original designation.
Coelopimpla Brèthes, 1916: 402. Type species: Coelopimpla amadei Brèthes, by original designation.
Liotheronia Enderlein, 1919: 147. Type species: Liotheronia kriegeri Enderlein, by original designation.
Dihyboplax Enderlein, 1919: 148. Type species: Dihyboplax flavipennis Enderlein, by original designation.
Neogabunia Brèthes, 1927: 322. Type species: Neogabunia paulistana Brèthes = Pimpla tomyris Schrottky, by monotypy.
Opodactyla Seyrig, 1932: 60. Type species: Pimpla (Opodactyla) waterloti Seyrig, by original designation.
Oxypimpla Noskiewicz & Chudoba, 1951: 42, 56. Type species: Pimpla turionellae Linnaeus, by monotypy.
Jamaicapimpla Mason, 1975. Type species: Ephialtes nigroaeneus Cushman, by original designation.

#### Diagnosis.

*Pimpla* can be distinguished from other genera of Pimplini (*sensu*Porter 1970 as *Coccygomimus*) by the combination of the following character states: 1) inner margin of eye weakly to rather strongly concave above antennal socket; 2) clypeus not divided by a transverse suture; 3) malar space 0.35–1.4 times as long as basal width of mandible; 4) mandible broad and with upper tooth approximately as long as the lower tooth; 5) notaulus weak or absent, without a distinct frontal crest; 6) propodeum with median longitudinal carinae varying from absent to sometimes weakly traceable throughout; 7) pleural carina usually present but sometimes absent; 8) length of fore wing 2.7–18.0 mm; 9) hind femur without a ventral tooth; 10) tarsal claws large and simple, without a basal lobe or an enlarged hair with a flattened tip; 11) metasoma varying from closely punctured to sometimes almost impunctate; 12) females with ovipositor approximately straight, ovipositor tip never sharply decurved.

Gauld et al. (2002) found a single autapomorphy for the genus: mid tarsomere IV medioventrally with a longitudinal band of fine hairs.

### Pimpla albomarginata

Taxon classificationAnimaliaHymenopteraIchneumonidae

Cameron, 1886

D6635216-C747-5D96-A7D2-A76991BE6E10

Figure 1A–F

Pimpla albo-marginata Cameron, 1886: 267. Holotype ♀, Mexico (BMNH).
Coccygomimus albomarginatus ; Townes and Townes 1966: 24.

#### Diagnosis.

This species can be distinguished from the other Uruguayan species of the genus by the combination of the following character states: 1) wings hyaline; 2) clypeus with apex deeply bilobed; 3) malar space wide, longer than basal mandibular width, that males less than 0.6 times basal mandibular width; 4) mesoscutum entirely black; 5) postscutellum black; 6) mesopleural suture weakly faveolated; 7) propodeum with conspicuous posterolateral tubercles; 8) fore wing Rs more or less straight and cu-a slightly distal to the base of Rs&M; 9) coxae without black markings and fore coxa with markings; 10) metasoma black and white banded; 11) laterotergites V broad, more than 0.5 times as broad as long; 12) tergite I of female short and broad, in profile strongly convex, in profile with moderately high blunt hump; 13) sternite I with strongly produced swelling; 14) apex of ovipositor with dorsal valve of ovipositor apically without teeth.

#### Biological notes.

Nothing is known about the host preferences of this species.

#### Distribution.

Colombia, Costa Rica, Mexico, Panama, Venezuela, and Uruguay* (Fig. 11A).

#### Material examined.

Uruguay, Rocha, Don Bosco, Bosque-Campo, 34°05'02.6"S, 53°45'44.5"W, 10.VI.2015, Malaise trap I (E. Castiglioni and team leg.), 1♀, INPA; Cardoso, Campo Natural, 34°05'28.0"S, 53°52'11.4"W, 10.VI.2015, Malaise trap II (E. Castiglioni and team leg.), 1♀, INPA; Don Bosco, Bosque-Campo, 34°05'02.6"S, 53°45'44.5"W, 12.I.2015, Malaise trap I (E. Castiglioni and team leg.), 1♂, INPA.

**Figure 1.**
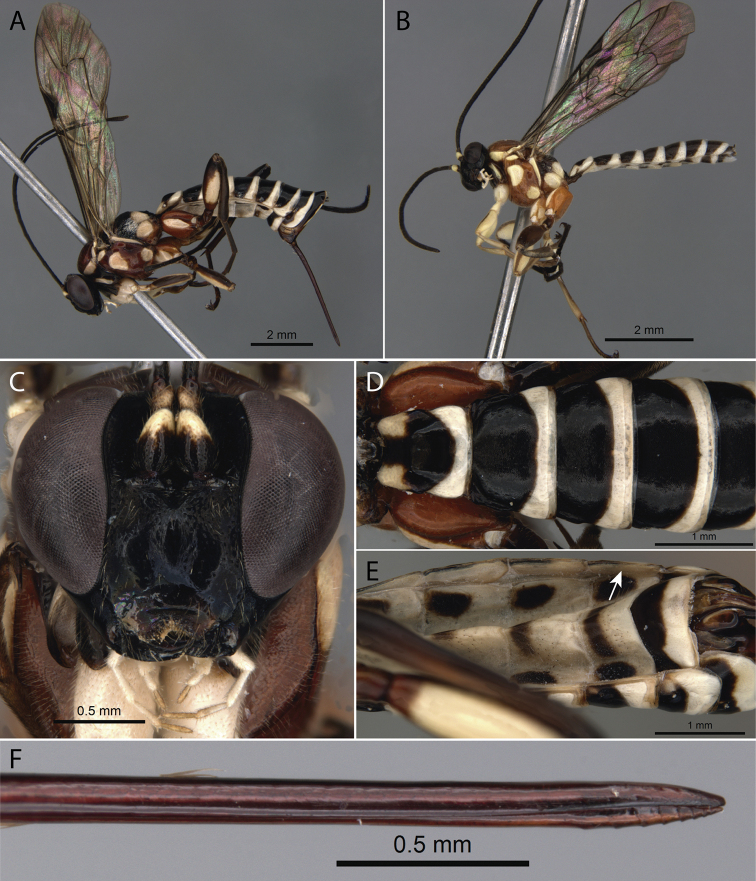
*Pimpla albomarginata* Cameron, 1886 **A** ♀, habitus, lateral view **B** ♂, habitus, lateral view **C** ♀, face, frontal view **D** ♀, metasoma, dorsal view **E** ♀, metasoma, ventral view (arrow pointing to laterotergite V) **F** ♀, ovipositor apex.

### Pimpla caerulea

Taxon classificationAnimaliaHymenopteraIchneumonidae

Brullé, 1846

D3672312-91C2-53C2-91AB-64E81B5DA178

Figure 2A–F

Pimpla caerulea Brullé, 1846: 101. Type: ♀, Brazil (MNHN).
Coccygomimus caeruleus caeruleus ; Townes and Townes 1966: 24.
Coccygomimus caeruleus glaucus ; Townes and Townes 1966: 25.

#### Diagnosis.

This species can be distinguished from the other Uruguayan species of the genus by the combination of the following character states: 1) wings more or less blackish; 2) body metallic blue (male with fore coxae white marked); 3) laterotergite V narrow, less than 0.3 times as long as wide.

#### Biological notes.

Parasitoid of *Alabama argillacea* (Hübner, 1818) (Noctuidae) (Porter 1970).

#### Distribution.

Argentina, Bolivia, Brazil, Ecuador, Guatemala, Mexico, Peru, Paraguay, Uruguay* (Fig. 11B), and Venezuela.

#### Material examined.

Uruguay, Rocha, Don Bosco, Bosque-Campo, 34°05'02.6"S, 53°45'44.5"W, 29.XII.2014, Malaise trap II (E. Castiglioni and team leg.), 1♀, INPA; idem, but 12.I.2015, Malaise trap II, 1♀ and 3♂♂, INPA; idem, but 12.III.2015, Malaise trap II, 1♂, INPA; idem, but 26.II.2015, Malaise trap II, 1♀, INPA; idem, but 28.I.2015, Malaise trap I, 1♂, INPA; idem, but 28.I.2015, Malaise trap II, 1♂, INPA; idem, but 29.XII.2014, Malaise trap I, 1♂, INPA; idem, but 29.XII.2014, Malaise trap II, 2♀♀ and 2♂♂, INPA.

**Figure 2.**
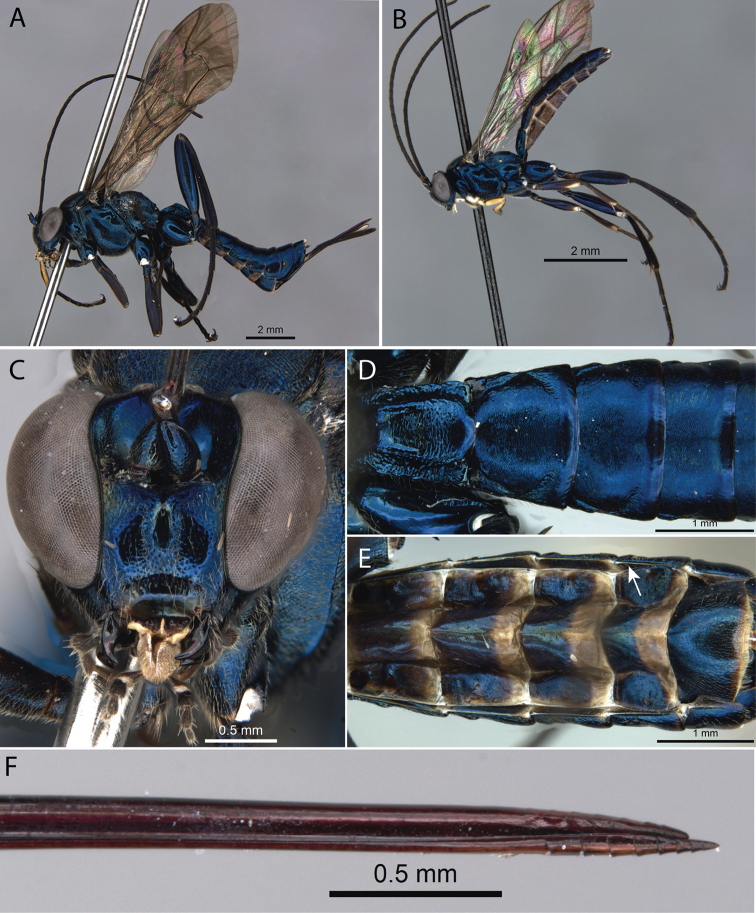
*Pimpla caerulea* Brullé, 1846 **A** ♀, habitus, lateral view **B** ♂, habitus, lateral view **C** ♀, face, frontal view **D** ♀, metasoma, dorsal view **E** ♀, metasoma, ventral view (arrow pointing to laterotergite V) **F** ♀, ovipositor apex.

### Pimpla cyanipennis

Taxon classificationAnimaliaHymenopteraIchneumonidae

Brullé, 1846

8DADFF5D-1FE5-5D84-B552-B38C9AF69496

Figures 3A–C , 4A–C

Pimpla cyanipennis Brullé, 1846: 101. Syntype: ♀, Uruguay (MNHN).
Coccygomimus cyanipennis ; Townes and Townes 1960: 328.

#### Diagnosis.

This species can be distinguished from the other Uruguayan species of the genus by the combination of the following character states: 1) wings darkened; 2) mesosoma and metasoma black; 3) laterotergite V 1.6–1.7 times as long as wide; 4) legs orange, except coxa, trochanter, trochantellus, apex of hind tibia and tarsus black; 5) tergite II silky shining, slightly coriaceous and mostly (except of apical rim), with almost uniformly distributed, large, strong, from more or less adjacent to confluent punctures; 6) malar space 1.0–1.2 times as long as basal width of mandibles; 7) ovipositor approx. 1.75 times as long as hind tibia; 8) ovipositor cylindric, with apex of dorsal valve without teeth and ventral valve with gently convex teeth on tip.

#### Biological notes.

Nothing is known about the host preferences of this species.

#### Distribution.

Argentina and Uruguay (Fig. 11C).

#### Material examined.

**Figure 3.**
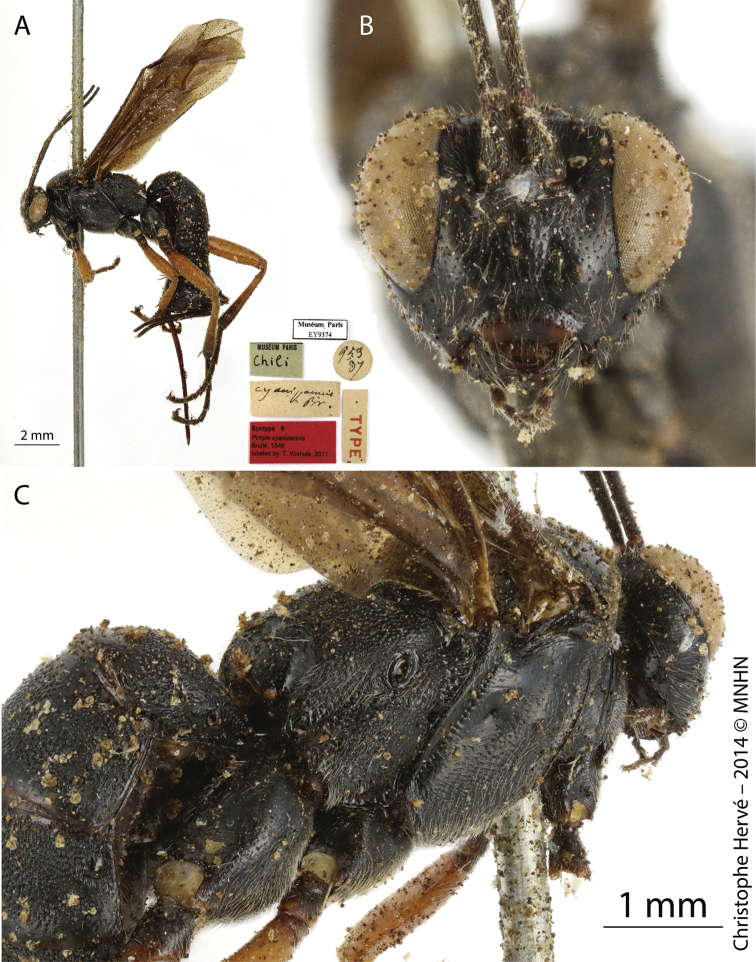
*Pimpla cyanipennis* Brullé, 1846 (Syntype, ♀) **A** habitus, lateral view **B** face, frontal view **C** mesosoma and part of metasoma, dorsolateral view. Figures by Christophe Hervé, MNHN.

**Figure 4.**
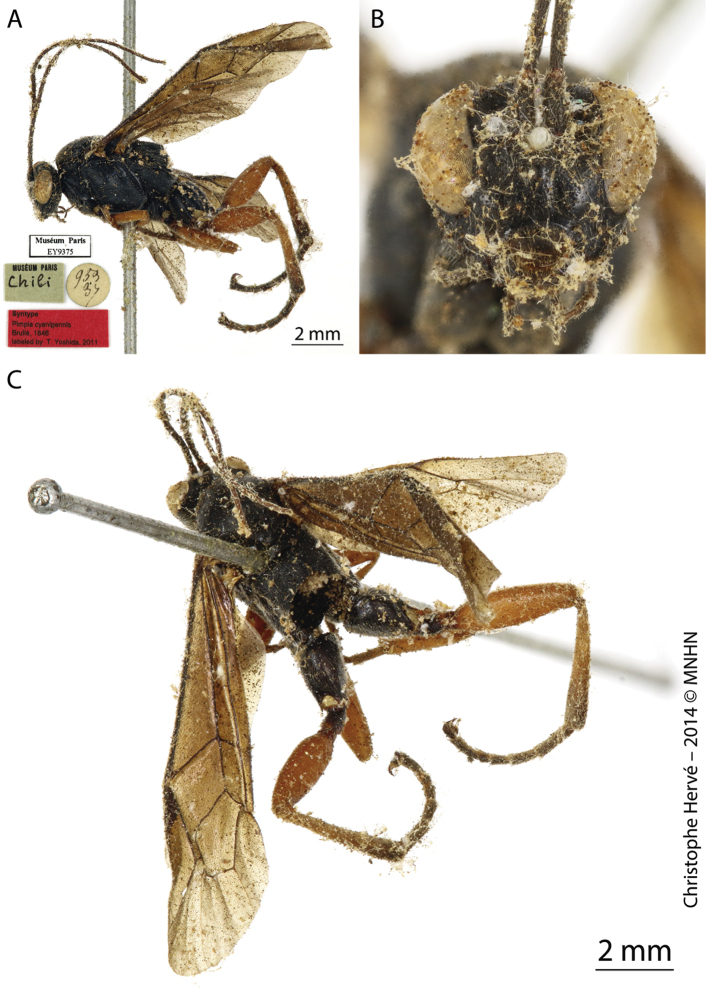
*Pimpla cyanipennis* Brullé, 1846 (Syntype, sex?) **A** habitus, lateral view **B** face, frontal view **C** mesosoma, dorsal view. Figures by Christophe Hervé, MNHN.

Syntype, Chile (♀, EY9374), examined by photo (Fig. 3A–C). Syntype, Chile (sex undetermined, EY9375), examined by photo (Fig. 4A–C).

#### Remarks.

Brullé (1846) described *P. cyanipennis* based on specimens from Montevideo (Uruguay; C. Gaudichaud collector). Later, Porter (1970) expanded the distribution of the species to Argentina. However, Porter did not study the type specimens of this species, deposited at MNHN. We analyzed the syntypes (EY9374 and EY9375), and verified that the type locality on the label is in Chile (C. Gay collector). The French botanist and naturalist Claude Gay carried out several expeditions in the Andes, especially in Chile and Peru. A large part of the material deposited by him in MNHN originated from these countries. Furthermore, Gaudichaud, who was appointed by Brullé as a collector of types, made several expeditions in Uruguay and Brazil (materials also deposited in MNHN). Thus, we hypothesize that: 1) the labels may have been unintentionally replaced in specimens, 2) the photos of the labels may have been added to the specimens in a wrong way in the MNHN database, or 3) Brullé may have confused the type locality when describing this species. Townes (1961) corrected inconsistencies in type localities in some species described by Brullé in MNHN, but he did not mention this species. In fact, we have studied the type specimens by using only photos, and we believe that only an *in situ* specimen examination can solve this inconsistency. Thus, we have decided to report this species only from Argentina and Uruguay.

### Pimpla golbachi

Taxon classificationAnimaliaHymenopteraIchneumonidae

(Porter, 1970)

EAD7A654-A251-5A7F-A72B-7401BBE30B5B

Figure 5A–F

Ephialtes kreibohmi Blanchard, 1942; *nomen nudum* according to Townes and Townes 1966: 29.
Coccygomimus golbachi Porter, 1970: 153. Holotype ♀, Argentina (IML).

#### Diagnosis.

This species can be distinguished from the other Uruguayan species of the genus by the combination of the following character states: 1) wings hyaline; 2) mesosoma black with hind corners of meso- and metapleuron brown and tegula white; 3) metasoma reddish; 4) laterotergite V 1.3 times as long as wide; 5) legs reddish, except of fore coxa often more or less broadly blackish basally, hind tibia sometimes slightly dusky, especially near apex, and tarsi usually duller often slightly dusky on apical segment; 6) tergite II shiny and with almost uniformly distributed large, deep, adjacent to reticulately confluent punctures, except narrowly smooth on apex; 7) malar space 0.8–1.0 (0.6–0.9 in male) times as long as basal width of mandibles; 8) ovipositor approx. 1.45–1.7 times as long as hind tibia; 9) ovipositor cylindric, dorsal valve with apex without teeth and ventral valve with gently convex teeth on tip.

#### Biological notes.

Parasitoid of Gelechiidae: *Pectinophora gossypiella* (Saunders, 1844); Noctuidae: *Alabama argillacea* (Hübner, 1818) (Porter 1970); Pieridae: *Colias lesbia* (Fabricius, 1775) (Avalos et al. 2011); Pyralidae: *Diaphania hyalinata* (Linnaeus, 1767); Tortricidae: *Rhyacionia buoliana* (Denis & Schiffermüller, 1775) (Porter 1970). Based on the material collected in our samples in Uruguay, we verified that the peak of occurrence of this species in the sampled locations was between November and January.

#### Distribution.

Argentina, Bolivia, Brazil, Colombia, Paraguay, and Uruguay (Fig. 11D).

#### Material examined.

Uruguay, Rocha, Castillos, Branaa, Agricultura, 34°03'31.8"S, 53°50'05.2"W, 30.XI.2015, Malaise trap II (E. Castiglioni and team leg.), 2♂♂, INPA; Castillos, Llambi, Pasto-agricultura, 34°24'7.04"S, 54°08'1.48"W, 12.II.2016, Malaise trap II (E. Castiglioni and team leg.), 1♀, INPA; idem, but 15.III.2016, Malaise trap II, 2♂♂, INPA; idem, but 28.I.2016, Malaise trap II, 1♀ and 1♂, INPA; Castillos, Cardoso, Campo Natural, 34°05'26.8"S, 53°52'14.4"W, 14.I.2016, Malaise trap I (E. Castiglioni and team leg.), 1♂, INPA; idem, but 15.III.2016, Malaise trap II, 1♀, INPA; idem, but 21.XII.2016, Malaise trap II, 1♂, INPA; idem, but 29.III.2016, Malaise trap I, 1♂, INPA; idem, but 29.III.2016, Malaise trap II, 1♂, INPA; idem, but 10.IV.2015, Malaise trap I, 1♀ and 1♂, INPA; idem, but 11.II.2015, Malaise trap II, 1♂, INPA; idem, but 12.I.2015, Malaise trap II, 1♂, INPA; idem, but 13.XI.2015, Malaise trap I, 4♂♂, INPA; idem, but 15.XII.2015, Malaise trap I, 1♂, INPA; idem, but 15.XII.2015, Malaise trap II, 1♂, INPA; idem, but 26.II.2015, Malaise trap II, 1♂, INPA; idem, but 26.V.2015, Malaise trap II, 1♂, INPA; idem, but 27.IV.2015, Malaise trap I, 1♀, INPA; idem, but 27.VII.2015, Malaise trap I, 1♀, INPA; idem, but 28.I.2015, Malaise trap II, 1♀, INPA; idem, but 29.XII.2015, Malaise trap I, 1♂, INPA; Castillos, Don Bosco, Bosque-Campo, 34°05'1.07"S, 53°45'43.08"W, 14.I.2016, Malaise trap I (E. Castiglioni and team leg.), 2♂♂, INPA; idem, but 14.I.2016, Malaise trap II, 1♀, INPA; idem, but 29.XII.2015, Malaise trap I, 1♀ and 2♂♂, INPA; idem, but 11.IX.2015, Malaise trap I, 1♀ and 1♂, INPA; idem, but 12.I.2015, Malaise trap I, 1♀ and 1♂, INPA; idem, but 12.I.2015, Malaise trap II, 1♂, INPA; idem, but 12.III.2015, Malaise trap I, 1♂, INPA; idem, but 13.XI.2015, Malaise trap I, 1♂, INPA; idem, but 13.XI.2015, Malaise trap II, 2♂♂, INPA; idem, but 15.XII.2015, Malaise trap I, 1♂, INPA; idem, but 15.XII.2015, Malaise trap II, 1♂, INPA; idem, but 27.X.2015, Malaise trap I, 1♂, INPA; idem, but 27.X.2015, Malaise trap II, 1♀, INPA; idem, but 28.I.2015, Malaise trap I, 2♂♂, INPA; idem, but 28.I.2015, Malaise trap II, 1♂, INPA; idem, but 28.IX.2015, Malaise trap I, 1♀, INPA; idem, but 29.XII.2014, Malaise trap I, 1♀ and 1♂, INPA; idem, but 29.XII.2014, Malaise trap II, 3♂♂, INPA; idem, but 30.XI.2015, Malaise trap I, 3♀♀, INPA.

#### Remarks.

Townes and Townes (1966) reported a new species of *Coccygomimus* as “*Coccygomimus* n. sp.” from Argentina and considered *Ephialtes kreibohmi* Blanchard, 1942 to be *nomen nudum* of it. Later, Porter (1970) described the species mentioned by Townes and Townes (1966) as *Coccygomimus golbachi*.

**Figure 5.**
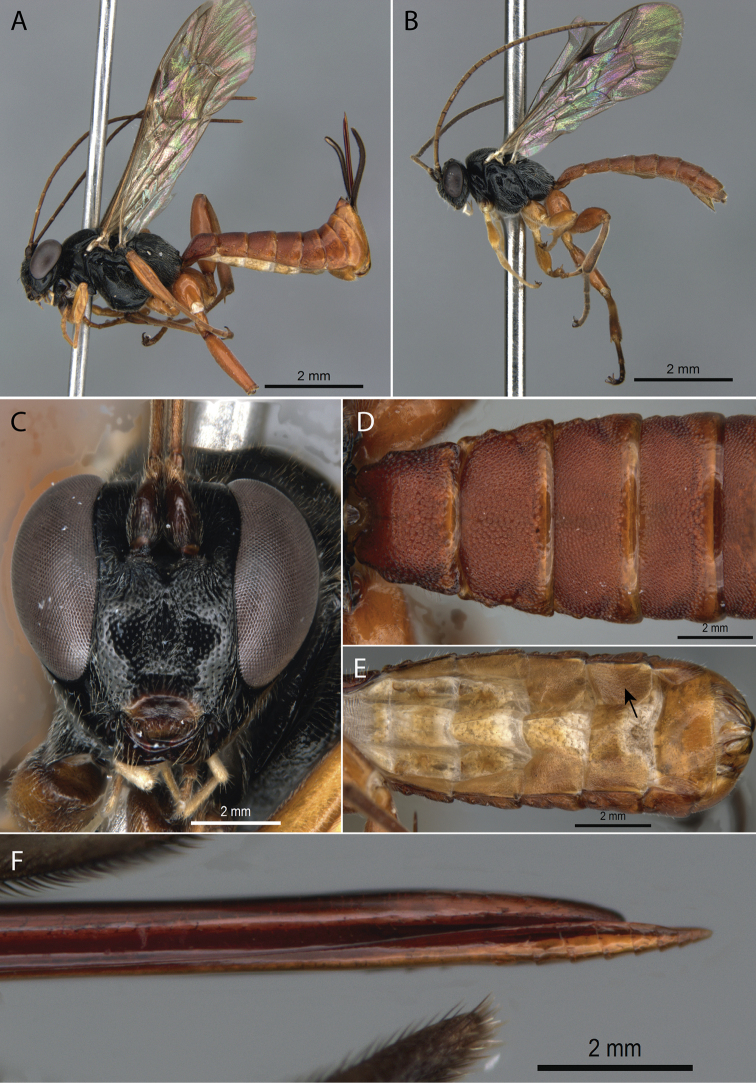
*Pimpla golbachi* (Porter, 1970) **A** ♀, habitus, lateral view **B** ♂, habitus, lateral view **C** ♀, face, frontal view **D** ♀, metasoma, dorsal view **E** ♀, metasoma, ventral view (arrow pointing to laterotergite V) **F** ♀, ovipositor apex.

### Pimpla patirrufa nom. nov.

Taxon classificationAnimaliaHymenopteraIchneumonidae

B6244DF8-DF13-514E-8C5D-02651C8016AE

Figures 6A–F , 7A–C

Pimpla rufipes Brullé, 1846: 102. Lectotype: ♀, Uruguay (MNHN). Non Pimpla rufipes (Miller, 1759).
Coccygomimus rufipes ; Townes and Townes 1960: 338.
Coccygomimus rufipes ; Townes 1961: 173.
Coccygomimus rufipes ; Townes and Townes 1966: 27.

#### Diagnosis.

This species can be distinguished from the other Uruguayan species of the genus by the combination of the following character states: 1) wings hyaline with weak brownish staining; 2) mesosoma shining black; 3) metasoma black with more or less brown staining on apical rims; 4) laterotergite V 1.4–1.6 times as long as wide; 5) legs orange with fore coxae orange or black, fore and mid tarsi slightly duller orange to slightly dusky, hind tibia duller orange with rather weak blackish staining on apex, hind tarsus extensively blackish to black; 6) tergite II with larger and stronger punctures; 7) malar space 1.0–1.1 (0.85–1.0 in male) times as long as basal width of mandibles; 8) ovipositor 1.3–1.6 times as long as hind tibia; 9) ovipositor cylindric, apex of dorsal valve without teeth and ventral valve with gently convex teeth on tip.

**Figure 6.**
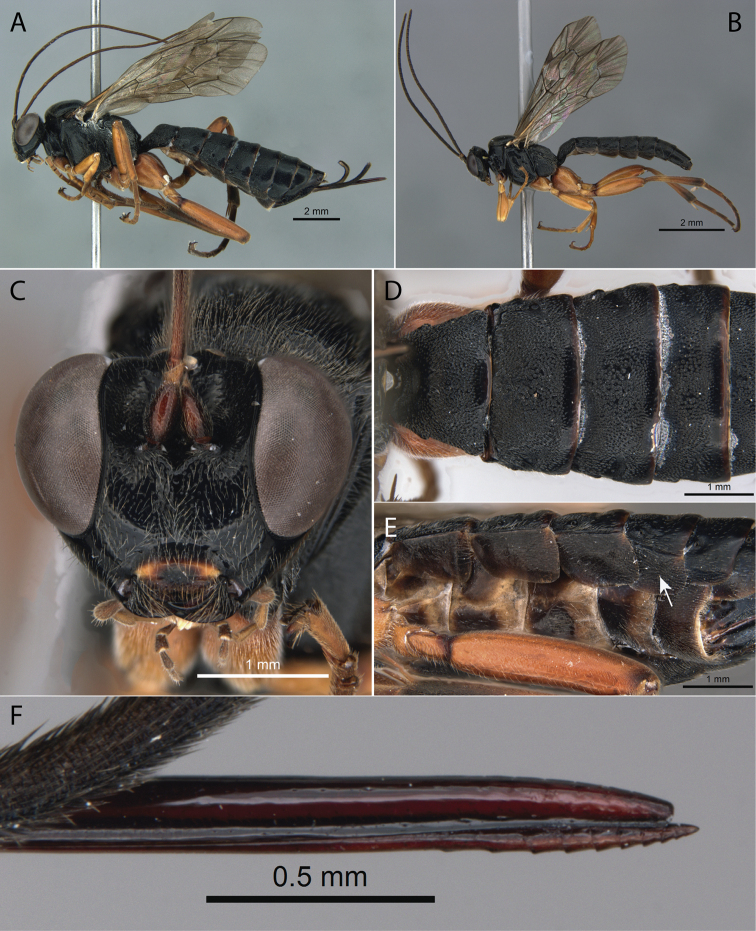
*Pimpla patirrufa* nom. nov. **A** ♀, habitus, lateral view **B** ♂, habitus, lateral view **C** ♀, face, frontal view **D** ♀, metasoma, dorsal view **E** ♀, metasoma, ventral view (arrow pointing to laterotergite V) **F** ♀, ovipositor apex.

#### Biological notes.

Parasitoid of *Plusia* sp. (Noctuidae) (Porter 1970).

#### Distribution.

Argentina, Brazil, and Uruguay (Fig. 11F).

#### Material examined.

***Lectotype***, Uruguay, Montevideo (♀, EY9414), examined by photo (Fig. 8A–C). URUGUAY, Rocha, Branaa, Agricultura, 34°02'33.7"S, 53°50'03.1"W, 11.II.2015, Malaise trap II (E. Castiglioni and team leg.), 1♂, INPA; idem, but 12.I.2015, Malaise trap I, 3♂♂, INPA; idem, but Malaise trap II, 1♀ and 1♂, INPA; idem, but 12.III.2015, Malaise trap II, 1♂, INPA; idem, but 27.IV.2015, Malaise trap I, 1♂, INPA; idem, but 28.I.2015, Malaise trap II, 2♂♂, INPA; idem, but 29.XII.2014, Malaise trap II, 1♀, INPA; idem, but 30.XI.2015, Malaise I, 1♂, INPA; Cardoso, Campo Natural, 34°05'26.8"S, 53°52'14.4"W, 12.I.2015, Malaise trap I (E. Castiglioni and team leg.), 1♂, INPA; idem, but 26.II.2015, Malaise trap I, 1♂, INPA; idem, but 28.I.2015, Malaise trap I, 1♂, INPA; idem, but 29.XII.2014, Malaise trap I, 1♂, INPA; idem, but 29.XII.2014, Malaise trap II, 1♂, INPA; idem but 21.XII.2016, Malaise trap II, 1♂, INPA; Castillos, Llambi, Pasto-agricultura, 34°24'7.04"S, 54°08'1.48"W, 08.XII.2016, Malaise trap I (E. Castiglioni and team leg.), 1♂, INPA; idem, but 21.XII.2016, Malaise trap I, 1♀, INPA; idem, but 26.II.2015, Malaise trap II, 1♂, INPA; Don Bosco, Bosque-Campo, 34°05'02.6"S, 53°45'44.5"W, 10.VI.2015, Malaise trap I (E. Castiglioni and team leg.), 1♂, INPA; idem, but 11.II.2015, Malaise trap I, 1♂, INPA; idem, but 26.II.2015, Malaise trap I, 1♂, INPA; idem, but 28.I.2015, Malaise trap II, 1♂, INPA; idem, but 29.XII.2014, Malaise trap I, 2♂♂, INPA; idem, but 29.XII.2014, Malaise trap II, 1♂, INPA.

#### Etymology.

The new specific name “patirrufa” is derived from the Spanish words “patas rufas”, and refers to the Spanish transliteration of “rufipes”, the original name proposed by Brullé. The name is to be treated as a noun in apposition.

#### Remarks.

*Pimpla patirrufa* nom. nov. is a replacement name for *P. rufipes* Brullé, 1846. The name “rufipes” was already occupied by *Pimpla rufipes* (Miller, 1759). According to the International Code Zoological Nomenclature, Article 57 (ICZN 1999), we propose a replacement name for this primary junior homonym. This homonymy may have caused some confusion in the literature. Çoruh and Kesdek (2008), Özbek and Çoruh (2012), and Çoruh et al. (2014) cited *P. rufipes* Brullé, 1846 from Turkey and Horstmann (2001) cited this species from Germany, but these authors most probably wanted to refer to *P. rufipes* (Miller, 1759). *Pimpla rufipes* (Miller, 1759) is a widespread Old World species (Yu et al. 2016). However, as *Coccygomimus instigator* (Fabricius, 1793) (currently junior synonym of *P. rufipes* (Miller, 1759), it was introduced at least five times between 1972 and 1978 to USA from Morocco, Yugoslavia, Iran, Poland and Romania (Coulson et al. 1986). Zwakhals (2005) listed some morphological differences of *P. rufipes* (Miller, 1759) in comparsion with other European species of *Pimpla*. Some of these characteristics assure us that this species is not closely related to *P. rufipes* Brullé, 1846, as pubescence is whitish and the coxae and trochanter are reddish in Brullé’s species and pubescence is fuscous and the coxae and trochanter are black in Miller’s species. In addition, *P. rufipes* Brullé, 1846 has a distribution restricted to South America (Argentina, Brazil, and Uruguay).

**Figure 7.**
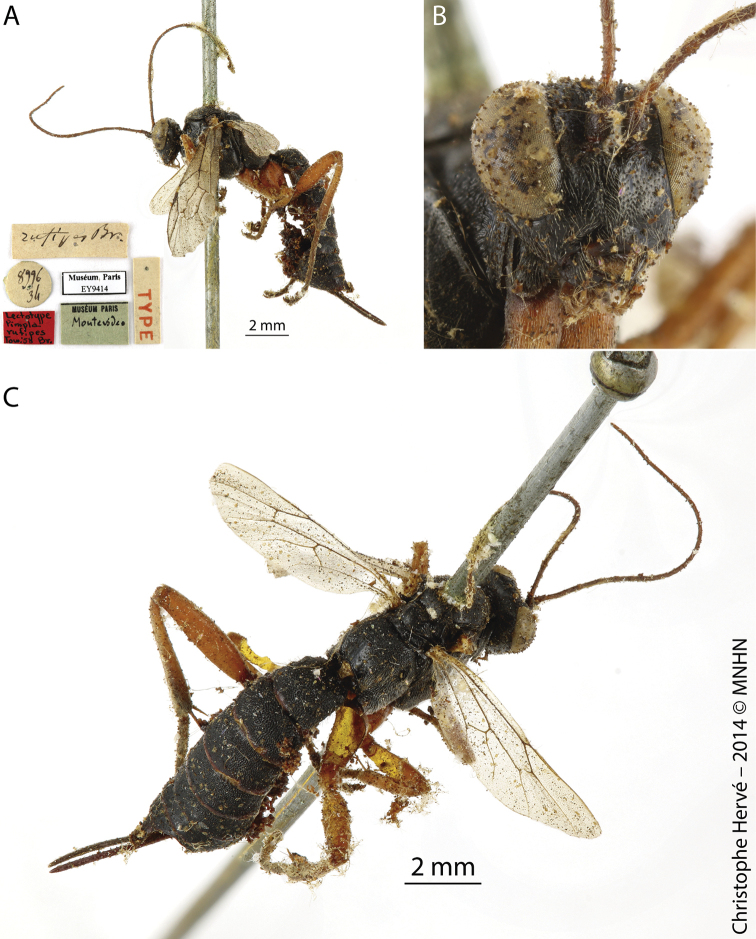
*Pimpla patirrufa* nom. nov. (Lectotype of *Pimpla rufipes* Brullé, 1846, ♀) **A** habitus, lateral view **B** face, frontal view **C** mesosoma and metasoma, dorsal view. Figures by Christophe Hervé, MNHN.

### Pimpla perssoni

Taxon classificationAnimaliaHymenopteraIchneumonidae

Gauld, 1991

B29532C8-9D05-580D-8116-DAB36A75BAA5

Figure 8A–F

Pimpla perssoni Gauld, 1991: 508. Holotype ♀, Costa Rica (MNCR).

#### Diagnosis.

This species can be distinguished from the other Uruguayan species of the genus by the combination of the following character states: 1) wings yellowish with distal margin of the fore wing blackish; 2) mesosoma yellow with black marks on mesoscutum (three stripes), hind part of tegula, hind margin of scutellum, anterior margin of mesopleuron, 7-shaped mark on upper hind part of mesopleuron, a continuous anterior band along the anterior margin of metapleura, and propodeum and hind rim of propodeum; 3) metasoma yellow with tergites I–IV anteriorly broadly and posteriorly narrowly black and with posterior tergites anteriorly black; 4) laterotergite V 2.7–3.4 times as long as wide; 5) legs yellow with dorsal longitudinal black band on med and hind coxa, fore, mid and hind (except the first tarsomere) tarsi strongly infuscate, femur darkened dorsally and ventrally, and tibia infuscate proximally, tibia with close and dark pubescence, giving them a dirty yellow appearance; 6) tergite II highly polished, with very fine sparse punctures, and with anterolateral corners separated by deep oblique grooves; 7) malar space 0.3–0.4 times as long as basal width of mandibles; 8) ovipositor 1.25–1.3 times as long as hind tibia; 9) apex of ovipositor slightly compressed, with weak denticles arranged in a median row on dorsal valve, and with ventral valve not expanded laterally, with a few weak teeth.

#### Biological notes.

Nothing is known about the host preferences of this species.

#### Distribution.

Brazil, Costa Rica, Mexico, and Uruguay* (Fig. 11E).

#### Material examined.

Uruguay, Rocha, Don Bosco, Bosque-Campo, 34°05'02.6"S, 53°45'44.5"W, 27.III.2015, Malaise trap II (E. Castiglioni and team leg.), 1♀, INPA; idem, but 28.I.2015, Malaise trap II, 1♂, INPA; idem, but 12.I.2015, Malaise trap II, 1♀, INPA; idem, but 29.XII.2014, Malaise trap I, 1♂, INPA; idem, but 12.I.2015, Malaise trap I, 1♂, INPA; idem, but 28.I.2015, Malaise trap I, 1♂, INPA.

**Figure 8.**
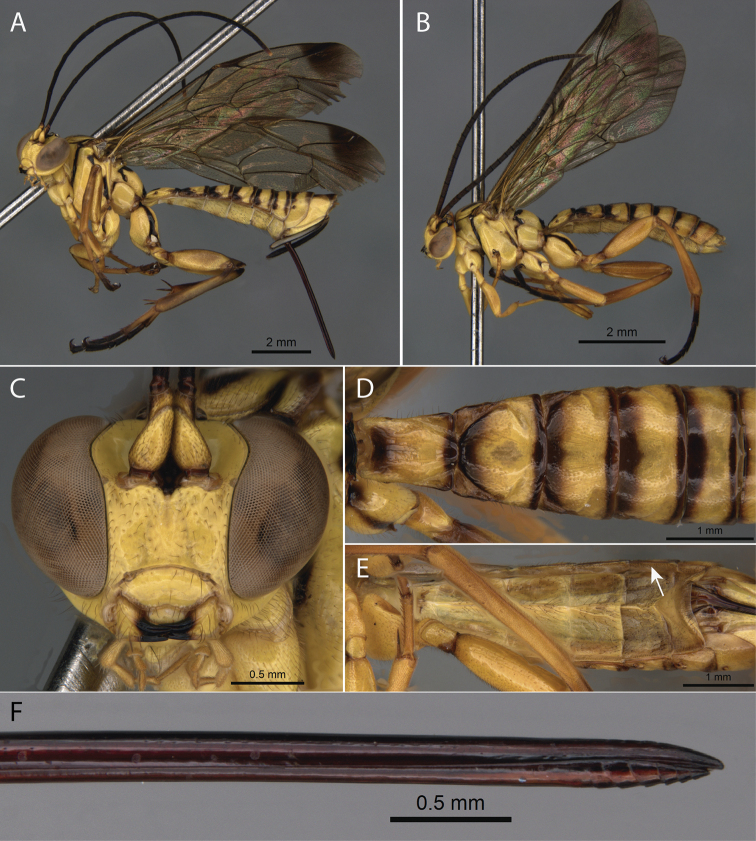
*Pimpla perssoni* Gauld, 1991 **A** ♀, habitus, lateral view **B** ♂, habitus, lateral view **C** ♀, face, frontal view **D** ♀, metasoma, dorsal view **E** ♀, metasoma, ventral view (arrow pointing to laterotergite V) **F** ♀, ovipositor apex.

### Pimpla semirufa

Taxon classificationAnimaliaHymenopteraIchneumonidae

Brullé, 1846

D5486D1B-2F17-5428-83C1-F57EBE936702

Figure 9A–F

Pimpla semirufa Brullé, 1846: 103. Type: ♀, Brazil (MNHN).
Coccygomimus semirufus ; Townes and Townes 1966: 28.

#### Diagnosis.

This species can be distinguished from the other Uruguayan species of the genus by the combination of the following character states: 1) wings hyaline; 2) mesosoma shining black, lower hind corner of mesopleuron slightly brown, metapleuron red with a little black staining along front margin and sometimes also along dorsal margin and propodeum red with slight to extensive black staining basad and in spiracular area; 3) metasoma reddish with tergite V often with a little blackish staining apico-laterally, tergite VI broadly to almost wholly black and tergites VII+ completely black; 4) laterotergite V 1.6 times as long as wide; 5) legs red, fore coxa black except becoming more or less broadly reddish toward apex below, fore and mid tarsi little duller reddish with slight dusky staining on apical segment, hind femur often with slight dusky tinge above on apex, hind tibia dull red basad and blackish on about apical half, hind tarsus dull red with much dusky staining; 6) tergite II shining with abundant, large, strong, mostly adjacent to confluent punctures, except on the narrow smooth apical rim; 7) malar space 0.8–1.0 times as long as basal width of mandibles; 8) ovipositor 1.3–1.6 times as long as hind tibia; 9) ovipositor cylindric, apex of dorsal valve without teeth and ventral valve with gently convex teeth on tip.

#### Biological notes.

Nothing is known about the host preferences of this species.

#### Distribution.

Argentina, Brazil, and Uruguay*(Fig. 11G).

#### Material examined.

Uruguay, Rocha, Cardoso, Campo Natural, 34°05'26.8"S, 53°52'14.4"W, 10.VI.2015, Malaise trap II (E. Castiglioni and team leg.), 1♀, INPA; idem, but 15.XII.2015, Malaise trap I, 1♂, INPA; idem, but 24.VI.2015, Malaise trap II, 1♂, INPA; Castillos, Don Bosco, Bosque-Campo, 34°05'1.07"S, 53°45'43.08"W, 21.XII.2016, Malaise trap I (E. Castiglioni and team leg.), 1♂, INPA; idem, but 21.XII.2016, Malaise trap I, 1♂, INPA; Castillos, Don Bosco, Bosque-Campo, 34°05'1.07"S, 53°45'43.08"W, 21.XII.2016, Malaise trap I (E. Castiglioni and team leg.), 2♂♂, INPA; Castillos, Llambi, Pasto-agricultura, 34°24'7.04"S, 54°08'1.48"W, 08.XII.2016, Malaise trap I (E. Castiglioni and team leg.), 1♂, INPA; idem, but 15.III.2016, Malaise trap II, 1♀, INPA; Don Bosco, Bosque-Campo, 34°05'02.6"S, 53°45'44.5"W, 10.VI.2015, Malaise trap I (Castiglioni and team leg.), 1♂, INPA; idem, but 12.I.2015, Malaise trap II, 2♂♂, INPA; idem, but 13.X.2015, Malaise trap II, 1♂, INPA; idem, but 14.I.2016, Malaise trap I, 2♂♂, INPA; idem, but 28.I.2015, Malaise trap I, 2♀♀, INPA; idem, but 29.XII.2014, Malaise trap II, 1♀, INPA.

**Figure 9.**
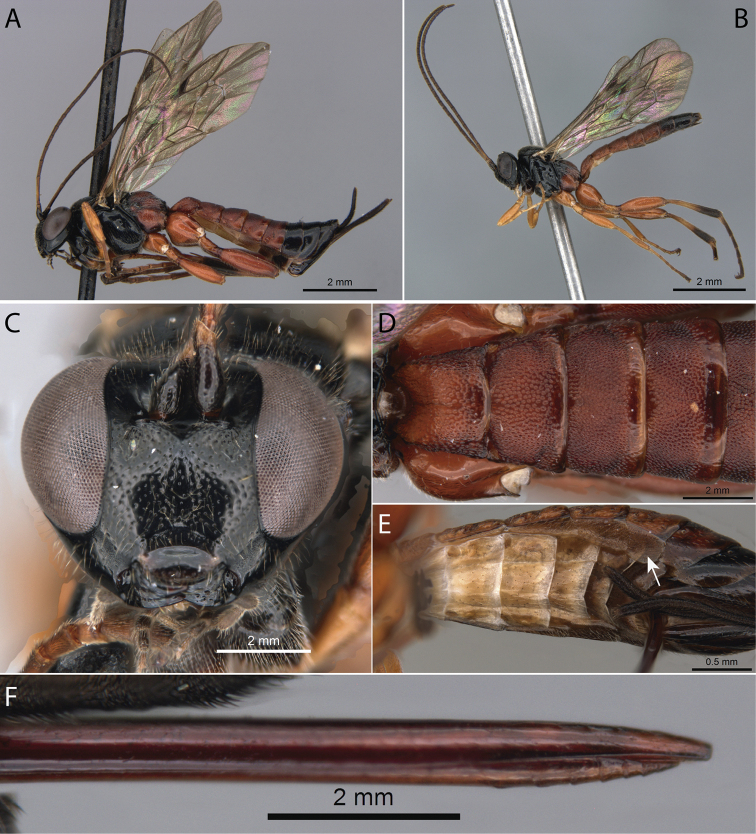
*Pimpla semirufa* Brullé, 1846 **A** ♀, habitus, lateral view **B** ♂, habitus, lateral view **C** ♀, face, frontal view **D** ♀, metasoma, dorsal view **E** ♀, metasoma, ventral view (arrow pointing to laterotergite V) **F** ♀, ovipositor apex.

### Pimpla tomyris

Taxon classificationAnimaliaHymenopteraIchneumonidae

Schrottky, 1902

2363D03F-F28F-5024-9305-BCBB64F38C6E

Figure 10A–F

Pimpla tomyris Schrottky, 1902: 95. Types: ♂, ♀, Argentina (lost).
Pimpla videonis ; Townes and Townes 1966: 28.
Neogabunia paulistana ; Townes and Townes 1966: 29.
Coccygomimus tomyris ; Townes and Townes 1966: 28.

#### Diagnosis.

This species can be distinguished from the other Uruguayan species of the genus by the combination of the following character states: 1) wings hyaline with pale yellow staining; 2) mesosoma shining black with variable yellow markings on pronotum, tegula, scutellum, postscutellum, and propodeum (a pair of elliptic blotches); 3) metasoma reddish brown with a pair of large yellow blotches laterally in tergites I–II (tergites I–IV in males); 4) laterotergite V 2.1–2.2 times as long as wide; 5) legs yellow, except for fore and mid coxa black (sometimes) and hind coxa with a black mark, femur and basal half of tibia reddish brown and last tarsus blackish; 6) tergite II rather dully to brightly shining with moderately strong to fine or very fine micro-reticulation and mostly sparse, irregularly spaced, small to large, obscure to well-defined punctures; 7) malar space 0.6–1.0 (0.4–0.7 in male) times as long as basal width of mandibles; 8) ovipositor 1.45 times as long as hind tibia; 9) ovipositor moderately depressed, apex of dorsal and ventral valves apically with teeth, the apical ridge-bearing portion not unusually flattened and in profile slightly convex.

**Figure 10.**
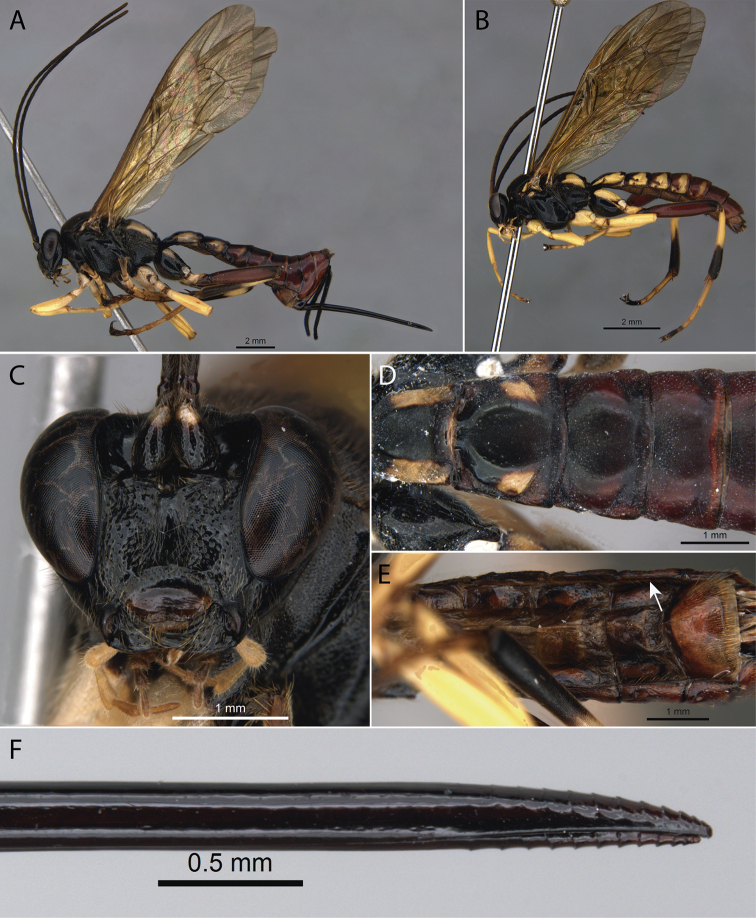
*Pimpla tomyris* Schrottky, 1902 **A** ♀, habitus, lateral view **B** ♂, habitus, lateral view **C** ♀, face, frontal view **D** ♀, metasoma, dorsal view **E** ♀, metasoma, ventral view (arrow pointing to laterotergite V) **F** ♀, ovipositor apex.

#### Biological notes.

Parasitoid of Erebidae: *Hypercompe indecisa* (Walker, 1855), *Hyposcrisias fuscipennis* (Burmeister, 1878); Limacodidae: *Phobetron hipparchia* (Crammer, 1777); Papilionidae: *Papilio thoas thoantiades* (Burmeister, 1878); Psychidae: *Oiketicus kirbyi* (Guilding, 1927), *O. platensis* (Berg, 1883); Saturniidae: *Eudyaria venata* (Butler, 1871), *Hylesia nigricans* (Berg, 1875); Tortricidae: *Rhyacionia buoliana* (Denis & Schiffermüller, 1775) (Yu et al. 2016).

#### Distribution.

**Figure 11.**
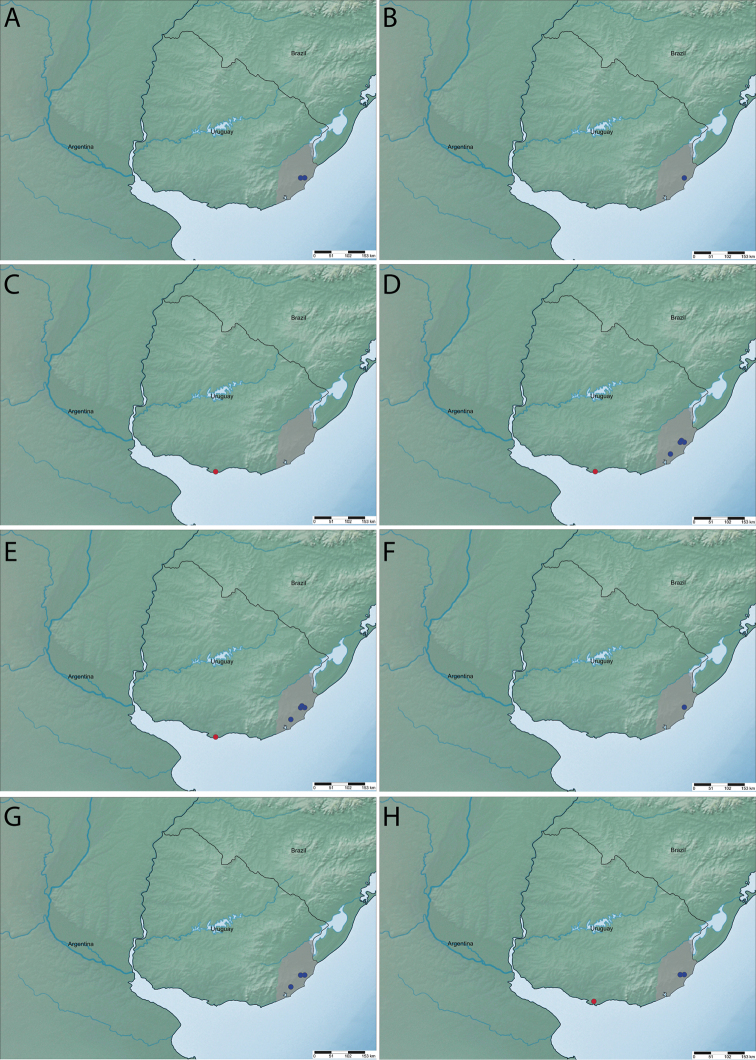
Distribution of *Pimpla* spp. in Uruguay **A***P. albomarginata* Cameron, 1886 **B***P. caerulea* Brullé, 1846 **C***P. cyanipennis* Brullé, 1846 **D***P. golbachi* (Porter, 1970) **E***P. perssoni* Gauld, 1991 **F***P. patirrufa* nom. nov. **G***P. semirufa* Brullé, 1846 **H***P. tomyris* Schrottky, 1902. Gray area = Rocha Department. Red circle = previous record. Blue circle = new record.

Argentina, Bolivia, Brazil, Paraguay, Peru, Uruguay, (Fig. 11H) and Venezuela.

#### Material examined.

Uruguay, Rocha, Castillos, Cardoso, Campo Natural, 34°05'26.8"S, 53°52'14.4"W, 28.XI.2016, Malaise trap I (E. Castiglioni and team leg.), 1♀, INPA; idem, but Don Bosco, Bosque-Campo, 34°05'02.6"S, 53°45'44.5"W, 12.I.2015, Malaise trap II, 1♂, INPA; idem, but except 28.I.2015, Malaise trap II, 1♂, INPA.

## Discussion

During the last 30 years, the Darwin wasp fauna of some Neotropical countries (i.e. Brazil, Costa Rica, and Peru) have been sampled in more detail. These studies have revealed a very high species richness and a plethora of new taxa from many parts of the region (e.g. Gauld 1991; Sääksjärvi et al. 2004; Veijalainen et al. 2012). However, most parts of the Neotropical region have remained understudied.

Uruguay’s biodiversity knowledge is still very fragmentary (Aldabe et al. 2008) and this is also shown by the genus *Pimpla*. Before of our study, only four species of *Pimpla* were known from the country: *P. cyanipennis* Brullé, 1846; *P. golbachi* (Porter, 1970); *P. patirrufa* nom. nov.; and *P. tomyris* Schrottky, 1902 (Yu et al. 2016). Here, we have doubled the species richness of *Pimpla* in Uruguay. All known Uruguayan species are also widely distributed in South America or the Neotropical region in general. *Pimpla* species are moderately large and strong-flying insects, which explains their wide distribution over vast regions.

We hope that this study draws more attention to Uruguay’s apparently rich, but very little-known, Darwin wasp fauna.

## Supplementary Material

XML Treatment for Pimpla

XML Treatment for Pimpla albomarginata

XML Treatment for Pimpla caerulea

XML Treatment for Pimpla cyanipennis

XML Treatment for Pimpla golbachi

XML Treatment for Pimpla patirrufa

XML Treatment for Pimpla perssoni

XML Treatment for Pimpla semirufa

XML Treatment for Pimpla tomyris
